# Supplementary material for: Chromosome-Level Comprehensive Genome of Mangrove Sediment-Derived Fungus Penicillium variabile HXQ-H-1
Source: J Fungi (Basel). 2019 Dec 23;6(1):7. doi: 10.3390/jof6010007 (PMC7151134; doi:10.3390/jof6010007)
Supplement: Supplementary file 1 [file jof-06-00007-s001.zip › Supplement Figures/Figure S2.pdf]

a

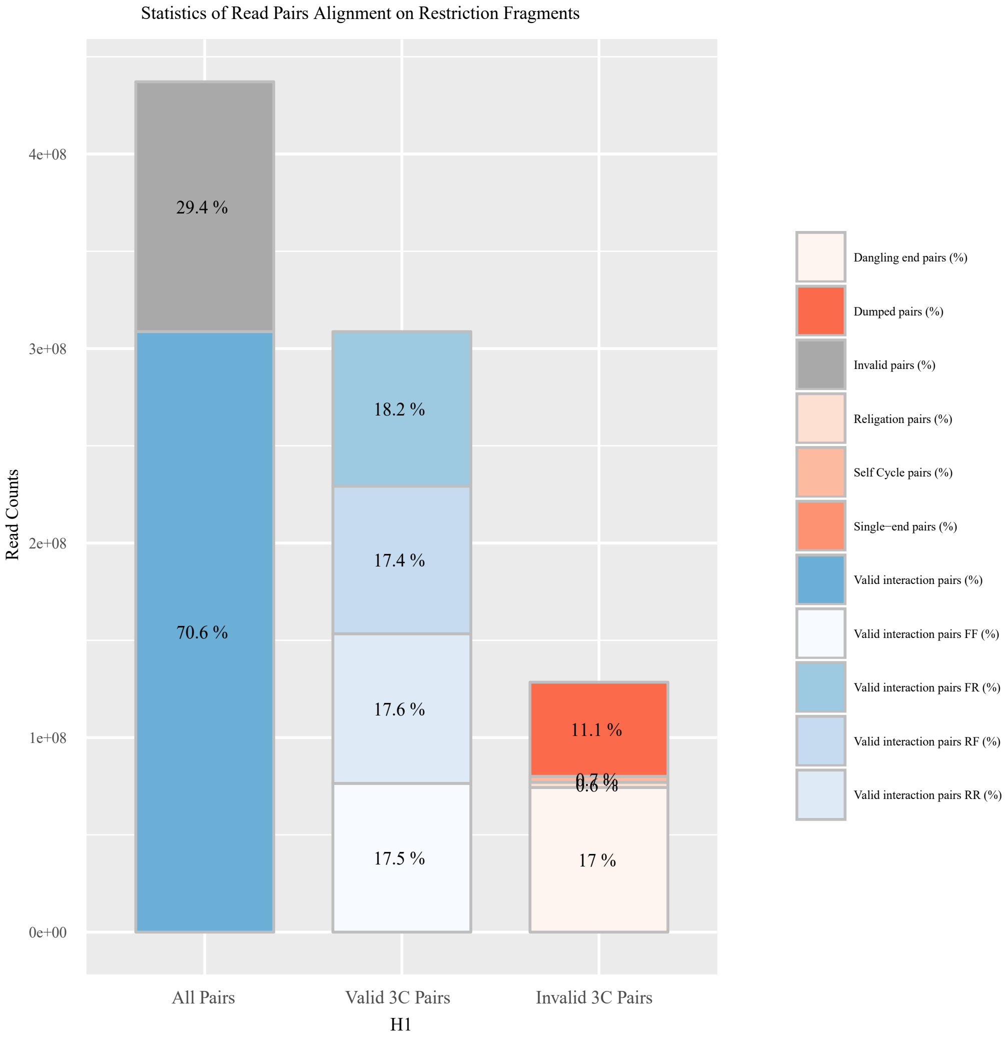

b

Type	Reads number
Valid interaction pairs	308,696,243
Valid interaction pairs FF	76,461,667
Valid interaction pairs RR	76,939,188
Valid interaction pairs RF	75,869,522
Valid interaction pairs FR	79,425,866
Dangling end pairs	74,339,487
Religation pairs	2,697,140
Self- Cycle pairs	3,060,122
Dumped pairs	48,361,073
