# Supplementary material for: Chromosome-Level Comprehensive Genome of Mangrove Sediment-Derived Fungus Penicillium variabile HXQ-H-1
Source: J Fungi (Basel). 2019 Dec 23;6(1):7. doi: 10.3390/jof6010007 (PMC7151134; doi:10.3390/jof6010007)
Supplement: Supplementary file 1 [file jof-06-00007-s001.zip › Supplementary Figure.docx]

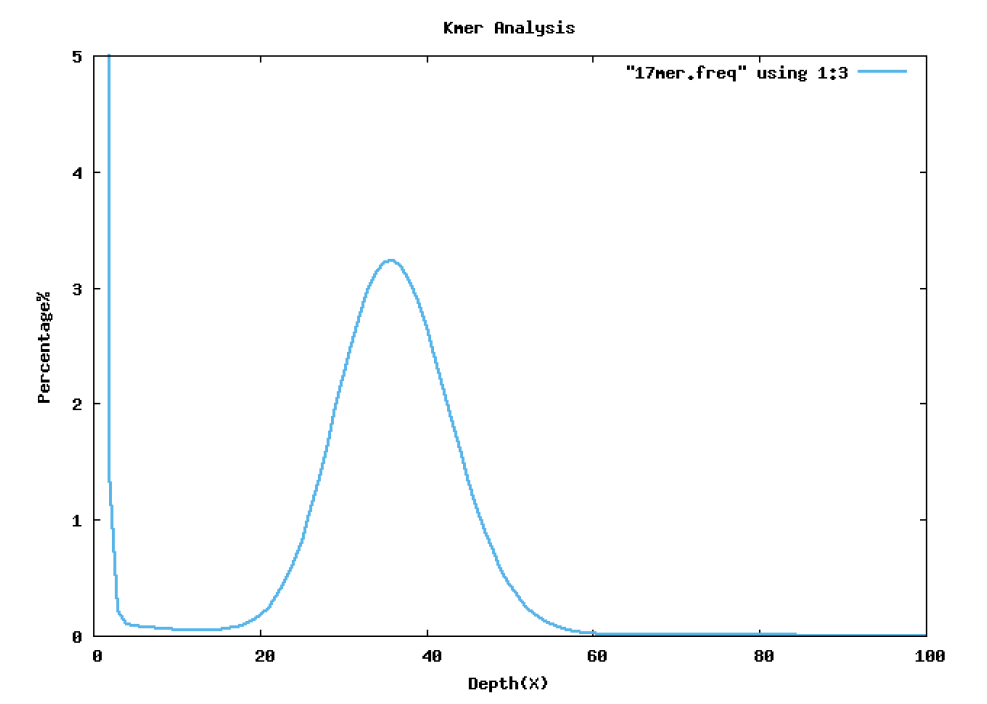


**Figure S1** 17-mers frequency distribution of HXQ-H-1. The x-axis is the occurrence of 17mer, the y-axis is the frequency of a certain depth.


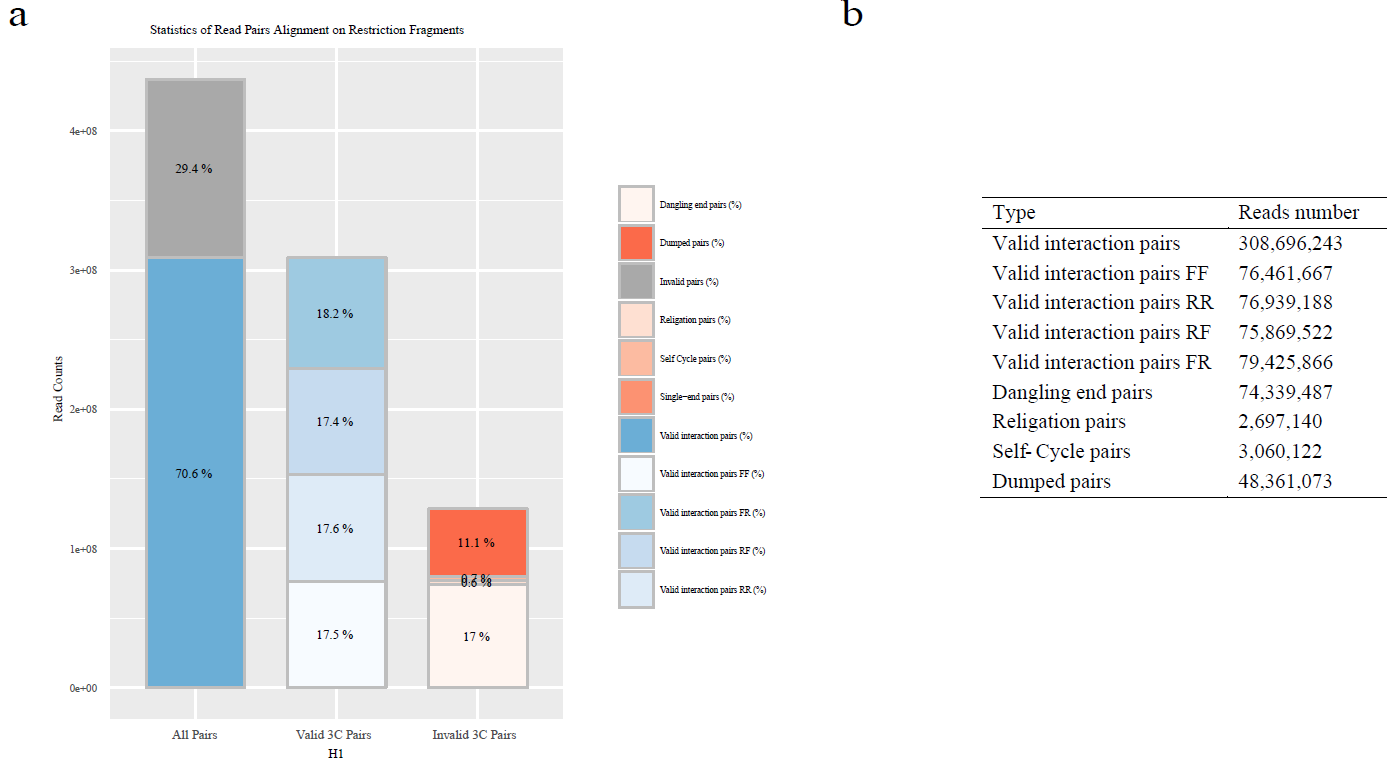


**Figure S2** Ratio of different types of reads qualified by HiC-Pro.


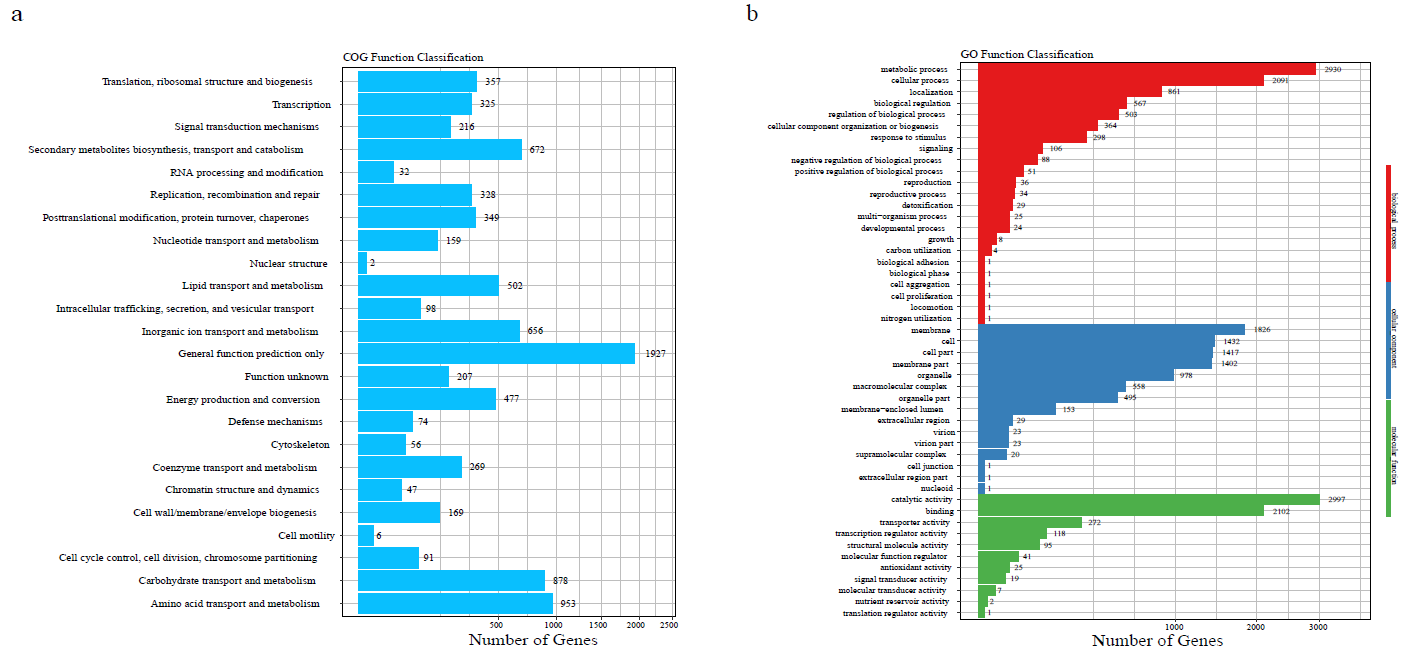


**Figure S3** Functional Categorization annotated by COG and GO database a). Histogram of COG annotation. b). Histogram of GO annotation. Biological process, cellular components and molecular functions are marked by different colors.
