# Supplementary figures and images for: Chromosome-Level Comprehensive Genome of Mangrove Sediment-Derived Fungus Penicillium variabile HXQ-H-1
Source: J Fungi (Basel). 2019 Dec 23;6(1):7. doi: 10.3390/jof6010007 (PMC7151134; doi:10.3390/jof6010007)

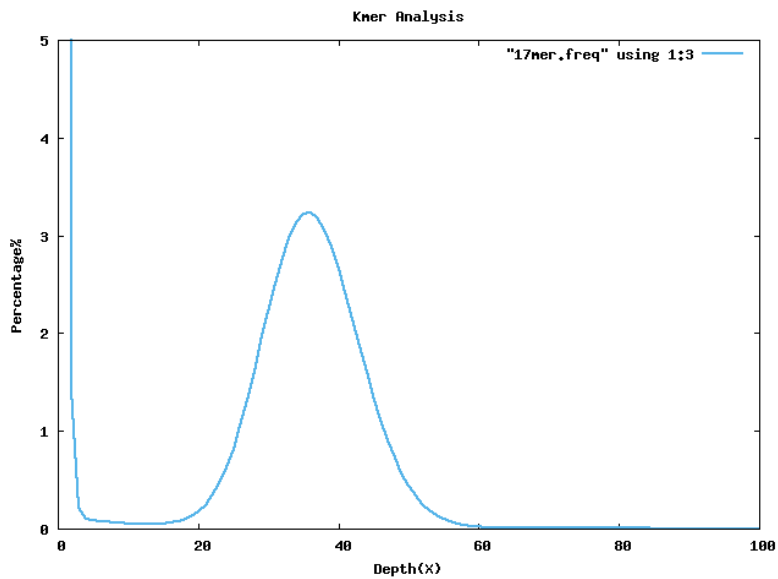

Supplement: Supplementary file 1 [file jof-06-00007-s001.zip › Supplement Figures/Figure S1.pdf]

a

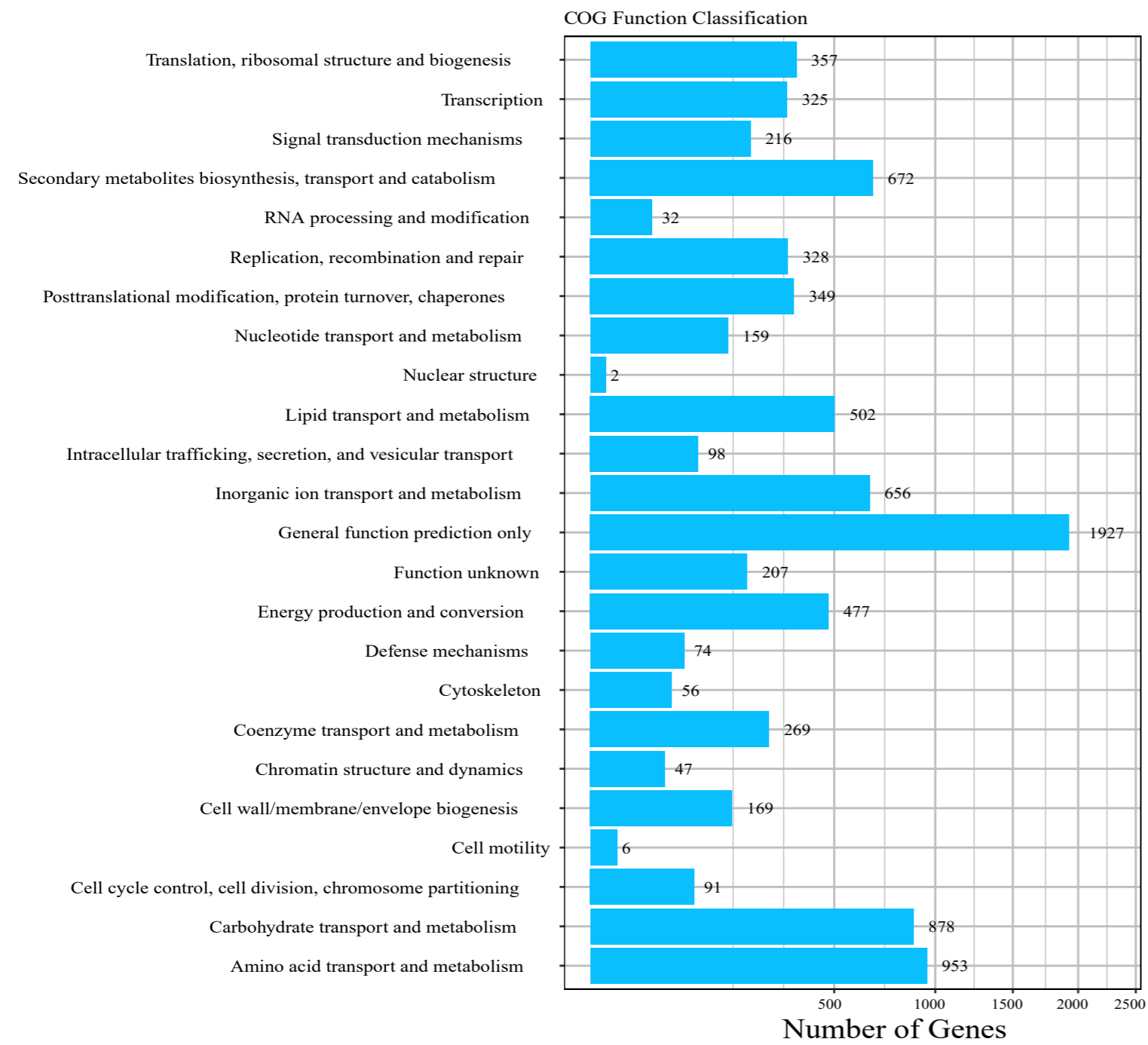

b

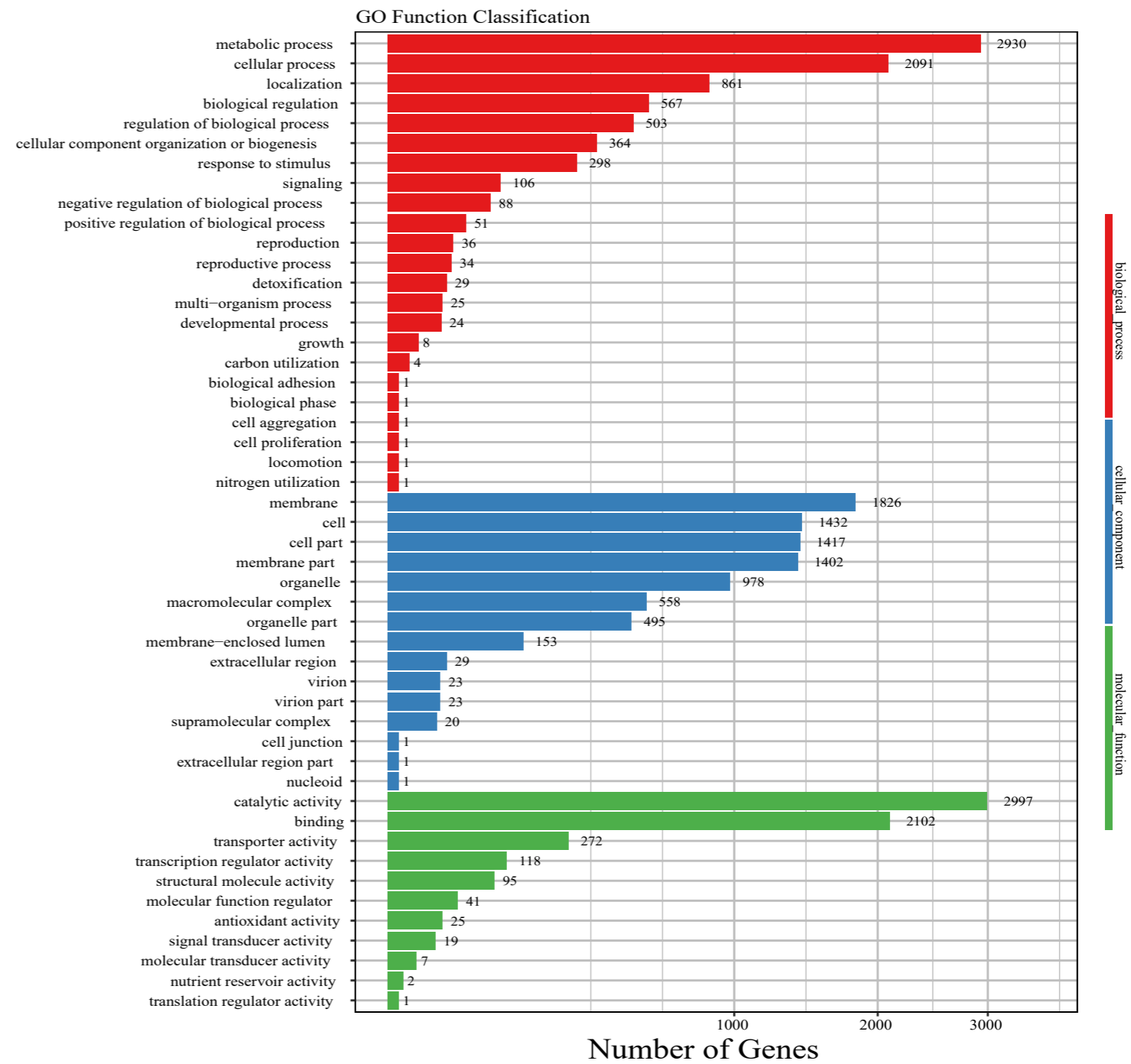

Supplement: Supplementary file 1 [file jof-06-00007-s001.zip › Supplement Figures/Figure S3.pdf]
